# Optical characteristics of highly conductive n-type GaN prepared by pulsed sputtering deposition

**DOI:** 10.1038/s41598-019-56306-0

**Published:** 2019-12-27

**Authors:** Kohei Ueno, Fudetani Taiga, Atsushi Kobayashi, Hiroshi Fujioka

**Affiliations:** 1 Institute of Industrial Science, The University of Tokyo, Meguro-ku, Tokyo, 153-8505 Japan; 2 ACCEL, Japan Science and Technology Agency (JST), Chiyoda-ku, Tokyo, 102-0075 Japan

**Keywords:** Semiconductors, Surfaces, interfaces and thin films

## Abstract

We have characterized highly conductive Si-doped GaN films with a high electron mobility of 112 cm^2^V^−1^s^−1^ at an electron concentration of 2.9 × 10^20^ cm^−3^, prepared using pulsed sputtering deposition (PSD). With an increase in the doping concentration, the absorption edge was found to shift toward a higher energy level, owing to the Burstein-Moss effect, thus making this material suitable for the transparent conductive tunneling electrodes of visible and ultraviolet-A light-emitting diodes. The full width at half maximum value of the near-band-edge (NBE) emissions in a photoluminescence spectrum measured at 77 K was as small as 185 meV, even for the sample with the highest electron concentration of 2.9 × 10^20^ cm^−3^. Such sharp NBE emissions from PSD-grown heavily Si-doped GaN films can be explained by an analytical model with a low compensation ratio *θ* of around 0.1, which is consistent with the exceptionally high observed electron mobility. These results indicate the strong potential of the low-temperature PSD growth technique for the formation of high-quality, heavily Si-doped GaN.

## Introduction

The preparation of high-quality heavily donor-doped n-type GaN is essential for reducing the parasitic resistance of various nitride devices such as light-emitting diodes (LEDs), laser diodes, and high electron mobility transistors. Heavy doping is especially important for the fabrication of the tunneling junctions used in optical devices. It is known that heavy n-type doping possibly modifies the fundamental properties of semiconductors owing to either their high concentration of impurities and/or free electrons. For wide-bandgap semiconductors such as GaN, the compensating effect has an important effect on their electrical and optical properties^1^. It is also well known that the formation energy of compensating point defects decreases with an increase in the Fermi level. The compensating defects act as effective scattering centers for electron transport and adversely affect the electron mobility in heavily n-type-doped GaN. This is one possible reason why the resistivity of n-type GaN films falls to a minimum value of 0.3–0.4 mΩ cm at electron concentrations of ca. 2 × 10^20^ cm^−3^ and increases with further increases in the dopant concentration^2,3^.

The random distribution of ionized donors as well as compensating acceptors also introduces potential fluctuations in the band edge, which affects the near-band-edge (NBE) emission and light-absorption processes. However, few systematic investigations have been undertaken on the impact of compensating point defects on the optical properties of heavily doped n-type GaN with electron concentrations of more than 1 × 10^20^ cm^−3^, as well as the relationship between the electrical and optical properties.

Recently, we found that the use of a sputtering process, named pulsed sputtering deposition (PSD)^4^, is advantageous for donor or acceptor doping in nitride films such as GaN and AlN with high carrier mobilities^5–9^. For PSD-grown Si-doped GaN, the minimum resistivity was found to be as low as 1.6 × 10^−4^ Ωcm with an electron concentration and mobility of 3.9 × 10^20^ cm^−3^ and 100 cm^2^V^−1^s^−1^, respectively^9^. This is the lowest resistivity attained for n-type GaN to date. Such high electron mobility can be explained by the low ionized impurity scattering rate caused by the low concentration of compensating levels. These achievements in the PSD growth of highly conductive Si-doped GaN films provide a good opportunity for the systematic investigation of the relationship between their optical and electrical properties.

In the present study, we investigated the optical properties of PSD-grown heavily Si-doped GaN films grown on ultraviolet-transparent AlN/sapphire templates using photoluminescence (PL) and transmittance measurements. We discussed the relationship between the electrical properties and optical properties, placing emphasis on the compensation ratio.

## Results and Discussion

**Figure 1:**
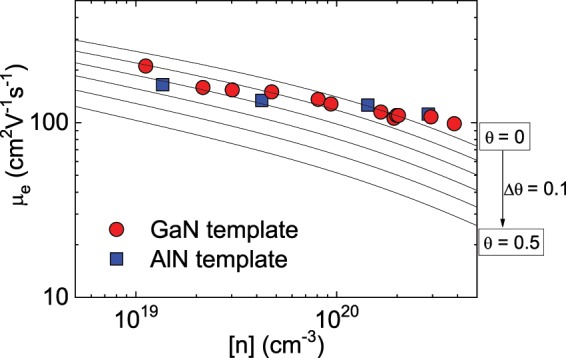
Measured electron mobility of heavily Si-doped GaN on AlN/sapphire templates as a function of electron concentration. The experimental data for the samples grown on GaN/sapphire templates and the calculated electron mobility for a compensation ratio (0 < θ < 0.5) were taken from our previous report^9^.

Figure 1 shows the RT electron mobility of highly Si-doped GaN grown on AlN/sapphire templates. In this figure, we also plotted the data for Si-doped GaN grown on GaN/sapphire templates and theoretically calculated mobility as a function of the compensation ratio *θ*, which were taken from our previous report^9^. The typical full width at half maximum (FWHM) values of the X-ray rocking curves of 0002 and 10$\bar{1}$2 diffraction were 300 and 2000 arcsec for the samples on the AlN/sapphire templates, respectively. Those for the samples on the GaN/sapphire templates were 300 and 400 arcsec. The corresponding dislocation densities were estimated to be 2 × 10^10^ cm^−2^ and 5 × 10^9^ cm^−2^ for the samples grown on the AlN/sapphire and GaN/sapphire templates, respectively. Despite the lower crystalline quality of the samples on the AlN/sapphire templates, their electron mobilities were as high as those of the samples grown on the GaN/sapphire templates. For example, the electron mobility remained as high as 112 cm^2^V^−1^s^−1^ at [n] = 2.9 × 10^20^ cm^−3^ for the samples on the AlN/sapphire templates. According to conventional scattering theory in heavily n-type doped GaN, the electron mobility is mainly limited by ionized impurity scattering because the dislocation scattering is well screened by the high density of the free electrons^10^. This result also indicates that spontaneous polarization charges at the heterointerface between the GaN and AlN have little impact on the electron transport properties in highly n-type doped GaN grown on the AlN/sapphire templates.

**Figure 2:**
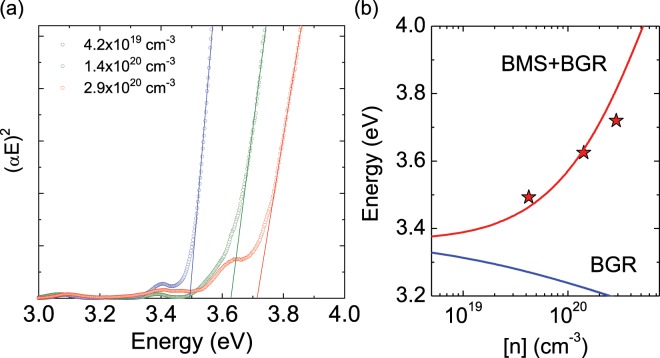
(**a**) Absorption spectra of heavily Si-doped GaN films grown on AlN/sapphire templates and (**b**) electron concentration dependence of optical bandgap.

The electron concentration dependence of the optical bandgap energy (*E*_opt_) of heavily Si-doped GaN was investigated by optical transmission measurement. The *E*_opt_ was determined from the Tauc plot, assuming direct transition as shown in Fig. 2(a). We can see that the absorption edge had shifted towards a higher energy level, from 3.49 to 3.72 eV, with an increase in the electron concentration. This phenomenon makes the heavily Si-doped PSD-GaN suitable for use as transparent conductive tunneling electrodes with p-type layers of visible and ultraviolet-A LEDs. The change in the *E*_opt_ of heavily doped semiconductors is usually explained by the Burstein-Moss shift (BMS) and bandgap renormalization (BGR), which cause the Fermi level to change in opposite directions. The BMS is related to the increase in the Fermi level, as is defined by $\Delta {E}_{BMS}=\frac{\hslash {k}_{F}^{2}}{2{m}_{e}}$, where *m*_e_ and *k*_F_ = (3*πn*)^1/3^ are the effective electron mass and Fermi wave vector, respectively^11^. The BGR was estimated using analytical expressions available in the literature^11^, and has contributions from both electron–electron and electron–ionized-impurity interactions. The analytical model of the relationship between *E*_opt_ and the electron concentration is depicted in Fig. 2(b). This is in good agreement with our experimental data. The slight deviation in the higher electron concentration may be related to the nonparabolicity of the conduction band.

**Figure 3:**
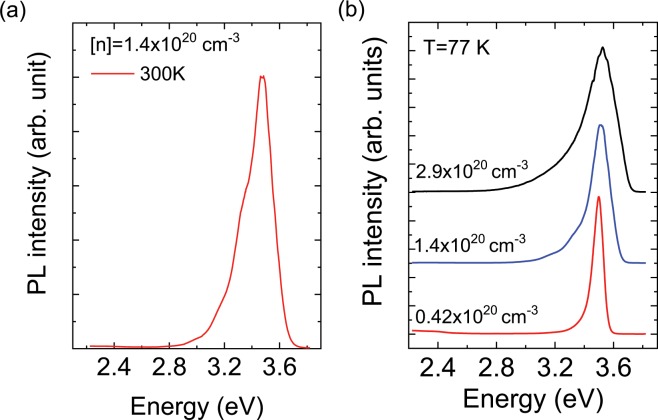
(**a**) Photoluminescence spectrum of heavily Si-doped GaN with [n] = 1.4 × 10^20^ cm^−3^ measured at 300 K. (**b**) Photoluminescence spectra for heavily Si-doped GaN films with different electron concentrations, measured at 77 K.

**Figure 4:**
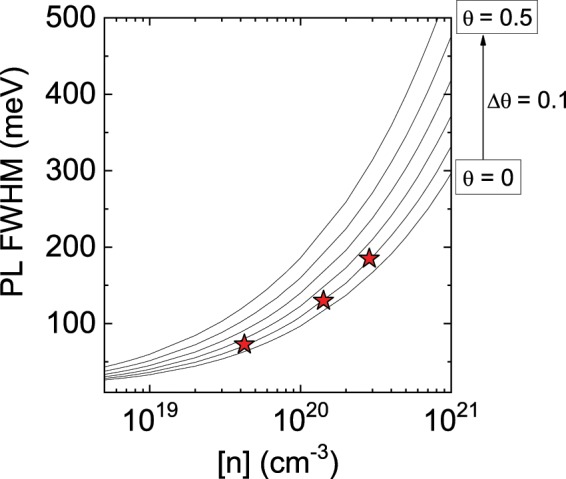
Experimental full width at half maximum values of near band edge emission measured at 77 K and analytical model curves for different compensation ratios^17^.

Figure 3(a) shows the PL spectrum of heavily Si-doped GaN with an electron concentration of 1.4 × 10^20^ cm^−3^ measured at 300 K. A strong near band edge (NBE) emission with a peak energy of 3.48 eV can be observed. The FWHM value was only 250 meV, even at this high electron concentration. Yoshikawa *et al*., proposed an empirical relationship between the FWHM value of the RT-PL emission and the electron concentration, which could be described by a *n*^2/3^ power-law dependence (49.7 meV + 8.7 × 10^12^ meVcm^2^ *n*^2/3^)^12^. Our experimental value was smaller than the calculated value of 284 meV, which indicates that the quality of the PSD-grown sample is high. The broad yellow luminescence (YL), which is usually assigned to carbon impurities or Ga vacancy (V_Ga_)-related defects^13,14^, was negligible. In fact, SIMS measurements revealed that the concentrations of the carbon atoms in the PSD-GaN films were very low and in the order of 10^15^ or 10^16^ cm^−3^. For the heavily Si-doped GaN grown by MOCVD, the YL intensity typically increased with the SiH_4_ flow rate and electron concentration, which is attributed to the formation of V_Ga_-O complexes^15^. First-principles calculations revealed that a higher Fermi level leads to a reduction in the formation energy of such defects^16^. On the other hand, the negligible YL intensity of our sample implied that the V_Ga_-related defect concentration should be much lower than that expected from the theoretical predictions, probably because our sputtering growth conditions were far from thermal equilibrium. Figure 3(b) shows the PL spectra measured at 77 K, for three samples with electron concentrations of 4.2 × 10^19^ cm^−3^, 1.4 × 10^20^ cm^−3^, and 2.9 × 10^20^ cm^−3^. The peak positions were shifted toward higher energy levels, from 3.50 to 3.53 eV, and their FWHM values also increased with the electron concentration. These emission peaks were associated with the direct transition between the conduction band and the valence band tail states. The broadening of the NBE emission line was attributed to the increase in the valence band edge potential fluctuations, resulting from the presence of impurities. Iliopoulos *et al*., proposed an analytical model for the width of the band tails and the electron concentration dependence of the PL FWHM values for n-type GaN^17^. In this model, randomly distributed ionized impurities screened by degenerate-free electrons give rise to the potential fluctuations. The presence of both donors and compensating acceptors was considered and thus the relationship between the PL FWHM values and the compensation ratio *θ* can be depicted as a function of the electron concentration. Figure 4 shows the measured FWHM values of the 77 K NBE emissions of our samples, as well as the analytical curves with different compensation ratios *θ*. The analytical PL FWHM values rapidly increased with the compensation ratio *θ* at higher doping levels. The measured FWHM value of the 77 K NBE mission was only 185 meV, even for the sample with the highest electron concentration of 2.9 × 10^20^ cm^−3^. Our experimental results were found to be in good agreement with the analytical model, assuming that the compensation ratio *θ* remains at low value of approximately 0.1, even for a high doping level. This assumption is quite consistent with the exceptionally high electron mobility of PSD GaN, discussed above.

## Conclusions

In conclusion, we used the PSD technique to grow highly conductive Si-doped GaN films on AlN/sapphire templates. The films yielded a maximum electron concentration of 2.9 × 10^20^ cm^−3^ with a high electron mobility of 112 cm^2^V^−1^s^−1^. The fact that the electron mobility of the heavily Si-doped GaN is not sensitive to the crystalline quality indicates that it was determined mainly by ionized impurity scattering, while the contribution from the threading dislocations is negligible. With an increase in the doping concentration, the absorption edge shifts towards a higher energy level due to the Burstein-Moss effect, which makes this material suitable for the transparent conductive tunneling electrodes of the p-type layers of visible and ultraviolet-A LEDs. The FWHM value of the NBE emission at 77 K was only 185 meV, even for a sample with the highest electron concentration of 2.9 × 10^20^ cm^−3^. Such a sharp NBE emission from PSD-grown highly Si-doped GaN films can be explained by the analytical model, assuming a low compensation ratio *θ* of around 0.1, which is consistent with the observed exceptionally high electron mobility. These results indicate the strong potential of the low-temperature PSD growth for the formation of high-quality heavily Si-doped GaN.

## Method

### Epitaxial growth

Heavily Si-doped GaN was grown on commercially available AlN/sapphire templates via PSD with pulsed magnetron sputtering sources in an N_2_/Ar atmosphere. The sputtering discharge power was set to be 80–100 W. The Si doping concentration in GaN was controlled by varying Si vapor flux from a solid state single crystalline Si source. The details of the growth procedure are available in the literature^5,7^.

### Electrical characterization

The electron concentration and mobility in the films were determined at room temperature (RT) by Hall effect measurements using the van der Pauw method. Ohmic contacts were then formed using a Ti/Al/Ti/Au (20/60/20/50 nm, respectively) stack as the electrodes. All the Hall-effect measurements were performed using a ResiTest 8400 device (Toyo Corporation).

### Optical characterization

For continuous-wave PL measurements, a HeCd laser (λ = 325 nm) was used as the excitation source. The PL signal was dispersed by a double monochromator (with a focal length of 0.75 m) with a 150-groove/mm grating. The dispersed signal was detected by an electronically cooled CCD allays. The transmittance measurements were carried out with a UV/Vis spectrometer V-570 (JASCO).
